# Risk of Adverse Events After Anti-TNF Treatment for Inflammatory Rheumatological Disease. A Meta-Analysis

**DOI:** 10.3389/fphar.2021.746396

**Published:** 2021-11-01

**Authors:** Ju Li, Zhongyuan Zhang, Xinhua Wu, Jie Zhou, Deqian Meng, Ping Zhu

**Affiliations:** ^1^ Department of Rheumatology, The Affiliated Huaian No.1 People’s Hospital of Nanjing Medical University, Huaian, China; ^2^ Department of Endocrinology, Huaian Hospital of Huaian City, Huaian, China

**Keywords:** anti TNF therapy, rheumatoid arthritis, psoriatic arthritis (artritis psoriatica), ankylosing spondylitis, risk of infections, malignancy

## Abstract

**Background:** Adalimumab, golimumab, infliximab, certolizumab, and etanercept are five anti-tumor necrosis factor (anti-TNF) medicines that have been approved for use in rheumatology. Apart from their well-established therapeutic usefulness, -it is unclear to what extent -they are linked to an increased risk of various side effects. The present meta-analysis was carried out to assess the risk of infection and other side effects after anti-TNF- α for the treatment of rheumatoid arthritis, psoriatic arthritis, and ankylosing spondylitis.

**Methods:** We searched PubMed, Cinahl (*via* Ebsco), Scopus, and Web of Sciences databases for trials comparing anti-TNF medications to placebo or no therapy in adult patients with rheumatoid arthritis, psoriatic arthritis, or ankylosing spondylitis from August 2006 to August 2020. A total of 23 articles were used for meta-analysis. The Cochrane Collaboration’s risk of bias tool was used to assess the methodological quality of the included studies. In addition, a random-effects model was used to calculate the pooled odds ratio, and Forest plots were constructed to determine the risk of infections and cancer following the use of anti-TNF treatment.

**Results:** Treatment with anti-TNFα agents resulted in an increase in the risk of serious infections (OR: 1.72, 95% CI: 1.56–1.90, *p* < 0.00001) and an increase in cancer risk (OR: 1.36, 95% CI: 1.20–1.53, *p* < 0.00001) whereas the risk of developing tuberculosis was not significantly different with anti-TNFα agents versus those without treatment with anti-TNFα agents (OR: 2.55, 95% CI: 0.40–16.23, *p* = 0.32) although the number of studies is limited to make a definitive conclusion. The risk of bias of the included studies was unclear to high across most domains, and there was evidence of publication bias for most outcomes.

**Conclusion:** The present meta-analysis suggests an increased risk of infectious adverse events, including overall adverse events and cancer following anti-TNFα treatment, whereas the risk of tuberculosis was not significantly different. Although anti-TNF agents have shown promise to treat inflammatory conditions, their use should be balanced by the risk-benefit ratio as suggested by the meta-analysis.

## Highlights

The present evidence suggests an increased risk of infections and malignancy after anti-TNF treatment. However, the risk of tuberculosis after anti-TNFα alpha therapy was not significant in the present meta-analysis. Therefore, it is essential to balance the risk-benefit profile when treatment with anti-TNFα is initiated in patients with inflammatory conditions.

Data on the development of adverse events following anti-TNFα treatment from data registries and surveillance reports is necessary to understand the long-term implications of treatment with biologic agents that extend beyond the time frame of randomized controlled trials.

## Introduction

Tumor necrosis factor (TNF) and interleukin-1 (IL-1) have been shown to play an essential role in the pathogenesis of inflammatory conditions such as rheumatoid arthritis (RA), psoriatic arthritis (PsA), and ankylosing spondylitis (AS) (Listing et al., 2005). Therefore, drugs targeting TNF and IL-1 have been developed to neutralize the effects of these pro-inflammatory cytokines. Five anti-TNF agents are currently available for clinical use in rheumatology: adalimumab, golimumab, infliximab, certolizumab, and etanercept. Adalimumab and golimumab are fully human monoclonal antibodies; infliximab is a chimeric monoclonal antibody with a murine variable region; certolizumab is a humanized Fab fragment conjugated with polyethylene glycol, while etanercept is a fusion protein of two TNF2 receptor extracellular domains and the Fc fragment of human immunoglobulin 1 (Curtis et al., 2007; Minozzi et al., 2016). Although these biologic agents differ in structure, they all act by neutralizing TNFα, which is implicated in early inflammatory events associated with several conditions. In addition, TNFα inhibitors have been used in rheumatological conditions that are unresponsive to treatment with disease-modifying anti-rheumatic drugs (DMARDs) such as sulfasalazine, chloroquine, hydroxychloroquine, D penicillamine, and azathioprine, among others. The Food and Drug Administration (FDA) has approved the use of biological TNFα blockers: Remicade^®^ (infliximab), Enbrel^®^ (etanercept), Humira^®^ (adalimumab), Cimzia^®^ (certolizumab pegol), and Simponi^®^ (golimumab).

Though several randomized controlled trials have successfully demonstrated the efficacy of these TNFα inhibitors, there is still debate regarding potential untoward effects of these biologicals upon long-term use. However, long-term use of anti-TNFα agents has been associated with the risk of serious infections, malignancies, skin and soft tissue infections, and tuberculosis (Baghai et al., 2001; Kroesen et al., 2003; Colombel et al., 2004). A meta-analysis carried out in 2006 showed an increase in the risk of malignancies and serious infections in patients treated with infliximab and adalimumab, where a higher dose was associated with increased cancer risk (Galloway et al., 2011). Although there has been no consensus on the risk of infections associated with anti-TNFα treatments in published clinical trials, post-marketing surveillance has shown an increase in the risk of tuberculosis and other granulomatous infections (Keane et al., 2001; Wallis et al., 2004). A recent meta-analysis by Bonovas et al. examined the effect of TNF inhibitors on the occurrence of malignancies in adult patients with RA, PsA, or AS. This study showed that there was no effect of anti-TNF agents on cancer risk in patients with RA, PsA, or AS using either fixed-effects or random-effects models. Furthermore, subgroup analysis according to the type of anti-TNF agent, did not demonstrate any statistically significant association between adalimumab, golimumab, infliximab, certolizumab, or etanercept and cancer risk.

Many countries have also developed national database registries that compile treatment outcomes and complications related to the prescribed treatment. These databases help in analyzing the safety of complications with biological therapies. However, many studies, including RCTs, case reports, etc., are emerging, stressing the incidence of various complications after receiving biological therapy for these disorders (Singh et al., 2009; Higgins et al., 2011; Abbott Laboratories, 2012; Downey, 2016).

The primary aim of this meta-analysis was to determine the relationship between anti-TNF α treatment versus no anti-TNF α treatment (or treatment with DMARDs) and risk of development of adverse effects such as serious infections, skin, and soft tissue infections and malignancies using data from randomized controlled trials (RCTs) and database registries in patients with rheumatoid arthritis, psoriatic arthritis, and ankylosing spondylitis.

## Materials and Methods

We followed the Preferred Reporting Items for Systematic Reviews and Meta-Analyses (PRISMA) normative recommendations in this study with the registration number NMU # RC/IRB/2020/3941.

### Data Search

From August 2006 to August 2020, a systematic search of PubMed, Cinahl (*via* Ebsco), Scopus, and Web of Sciences bibliographic databases was undertaken. Anti-tumor necrosis factor, tumor necrosis factor(s), tumor necrosis factor-alpha antibody (ies), tumo(u)r necrosis factor antibody (ies), anti-TNF, TNF, biologic (al) agent(s), or biologic(s), combined with rheumatoid arthritis, psoriatic arthritis, or ankylosing spondylitis. The search was restricted to observational studies, RCTs, and data from disease registries involving human participants. There were no limitations on grammar, date, or publishing status. We also looked through the Cochrane Library for any observational studies and RCTs that were part of the Cochrane Central Register of Controlled Trials, as well as any systematic reviews on the subject.

Two analysts (JL and ZZ) reviewed the search results separately and screened the titles and abstracts to exclude papers that were simply unrelated. The full text of the selected papers was scrutinized for eligibility. Their reference lists (and those of related reviews and meta-analyses) were manually checked for additional studies. Experts were surveyed for additional evidence, but no unpublished research or results were sought.

### Data Extraction

Observational Studies and RCTs evaluating an anti-TNF agent (adalimumab, certolizumab, etanercept, golimumab, or infliximab) as induction or maintenance therapy for adults with RA, PsA, or AS and reporting the presence of infectious adverse events and any type of cancer were considered. Any bacteria, severe infections (infections requiring antimicrobial treatment and/or hospitalization), cancer, tuberculosis, or opportunistic infections were all eligible outcomes. In addition, we looked for trials that linked an anti-TNF therapy to placebo or no medication, as well as multi-interventional treatments where the anti-TNF treatment effect could be isolated (i.e., an add-on to conventional disease-modifying anti-rheumatic drugs).

### Eligibility of Criteria

The articles were reviewed from the title or abstract. Case reports, adult studies, reviews, and editorial articles were excluded. However, articles concerning adults with at least one defined treatment group addressed various adverse effects written in English.

Information on the following aspects were included in the meta-analysis: risk of serious infections, risk of skin and soft tissue infections, tuberculosis, and cancer following treatment with anti-TNFα agents compared to non-biologics.

**FIGURE 1:**
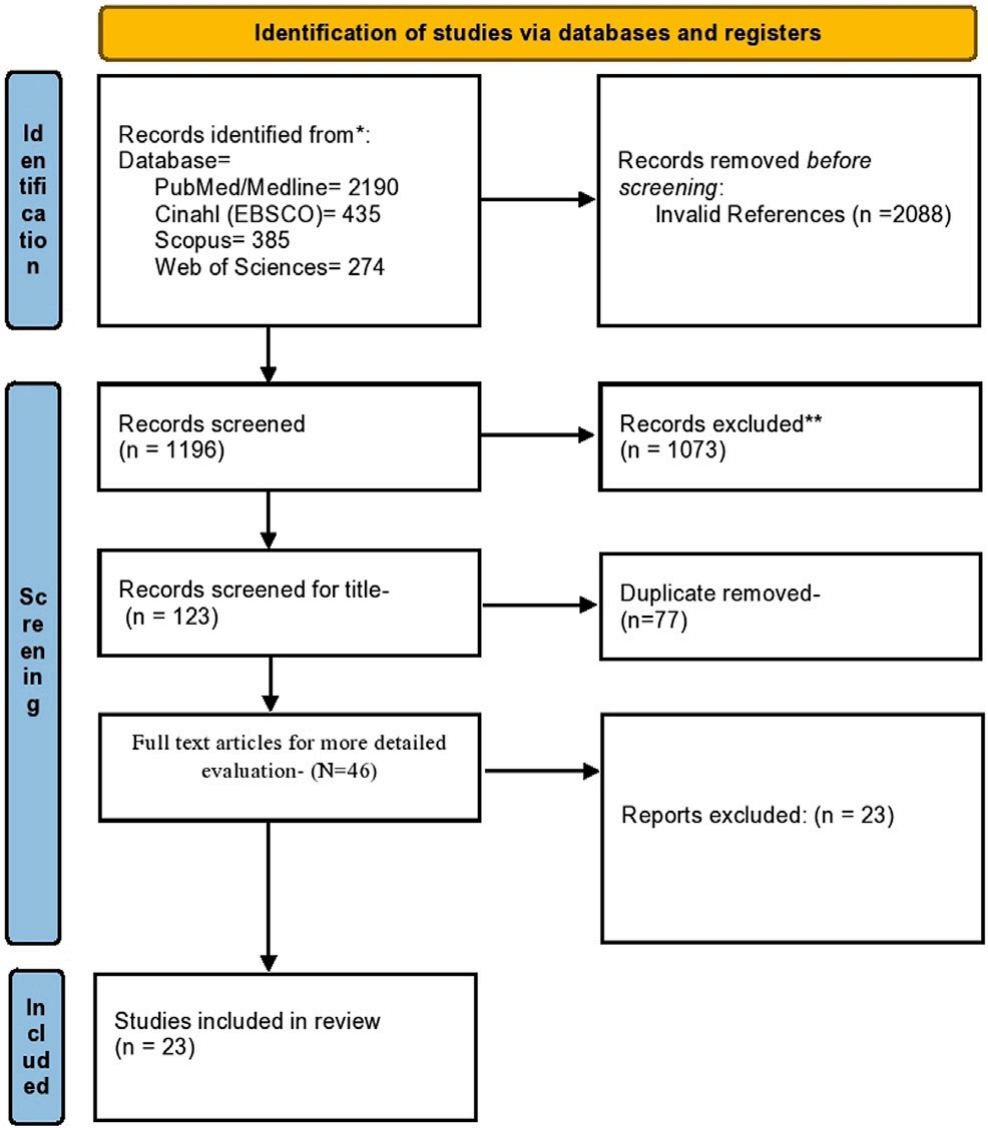
Flow chart for identification and inclusion of studies in the meta-analysis (PRISMA statement).

The primary search yielded 123 results. The articles were excluded based on the exclusion criteria. These included the 46 articles, out of which 23 articles were used for data analysis. The article search was limited to the English language, and no other limiting factor was used in finalizing the study. The details of the number of articles included are given in Figure 1.

When there were several papers from the same sample, we chose the most recent one and extracted the results over the longest possible period. Independent researchers oversaw data extraction. Any inconsistencies were overcome by consensus regarding the original paper. In addition, each study’s first author’s last name, journal and year of publication, trial acronym, study design and duration, number of participants, disease studied (RA, PsA, or AS), patient characteristics (age, concurrent treatments, disease duration), intervention parameters (drug, dose, administration), and numbers of participants with events (serious infections) were extracted.

### Quality Assessment

The Cochrane Collaboration’s risk of bias tool was used to assess the methodological quality of the included studies (Higgins et al., 2011). This tool includes the following criteria: randomization, allocation concealment, blinding, and completeness of follow-up. In addition, the risk of bias for each item was graded as high, low, or unclear risk.

### Statistical Analysis

Meta-analysis was performed using Review Manager (RevMan, Version 5. Copenhagen: The Nordic Cochrane Center, The Cochrane Collaboration. 2020). The total number of participants in each study developing an adverse event and the total number of participants in each group (intervention: anti-TNFα and control group: without anti-TNFα treatment) were used to calculate the odds ratio 95% confidence interval (CI). Meta-analyses were done using a random-effects model (Mantel-Haenszel method), and heterogeneity in the included studies was evaluated using *the I* ^*2*^ statistic, with small heterogeneity for *I* ^*2*^ values of 25%, moderate heterogeneity for *I* ^*2*^ values of 25–50%, and high heterogeneity for *I* ^*2*^ values >50% (Higgins et al., 2003). Forest plots were constructed, and *p* < 0.05 was statistically significant.

### Evaluation of the Publication Bias

Begg’s Funnel plot was used to determine the publication bias of the included studies. The methodological validity of the included studies was evaluated by two reviewers (JL and ZZ) separately. XW and JZ were in charge of resolving any disagreements between authors.

## Results

### Literature Search Results

Through electronic scans, we found a total of 3284 studies. By reading titles and abstracts, we excluded 1,073 on reading titles and abstracts and 2088 invalid references. Out of 123 studies, around 77 studies were excluded based on duplicity. Full-text publications were required for final screening was 46, of which 23 were excluded based on the inclusion criteria. Thus, the study and meta-analysis contained 23 studies that met the inclusion criteria, i.e., based on the adverse events associated with anti-TNF treatment, as shown in Figure 1. Inappropriate comparison criteria and inadequate evidence to create 2 × 2 tables for review were the key reasons for the omission.

Table 1 shows the demographic details of the studies included in the present meta-analysis describing the study author, year of publishing, place where the study was conducted, total sample size with age, the individual sample size in each group using anti-TNF agents compared to non-biologic, type of adverse events reported in groups using anti-TNFα agents and controlled or placebo, and key findings of all the included study. All studies were released as full-text papers. Infliximab (*n = 12*), adalimumab (*n = 18*), golimumab (*n = 10*), certolizumab pegol (*n = 8*), or etanercept (*n = 11*) is tested as induction or maintenance therapies for adult patients with RA (*n* = 38), PsA (*n* = 6), or AS (*n* = 8) in these 23tudies. A total of 75,406 patients were included in the present meta-analysis.

**TABLE 1:** Demographic Details of Included Studies.

Studies with year	Place	Study design	Sample size	Anti-TNF agent	Inflammatory condition	Follow-up	Mean age (yrs)	Sample size in anti TNF-α v/s controlled or placebo		Adverse events reported	Sample size with adverse events		Odds ratio (95% CI)
								Anti TNF-α	Without anti TNF-α		Anti TNF-α	Without anti TNF-α	
Galloway et al. (2011)	United Kingdom	Prospective Observational Study	15,396	Adalimumab, etanercept, infliximab	RA	6 months	58 years	11,798	3598	Serious Infection	1,512	296	1.64 (1.44–1.87)
Curtis et al. (2007)	United States	Retrospective Cohort Study	5,326	Adalimumab, etanercept, infliximab	RA	17 months	52.5 ± 12.5	2,393	2,933	Serious bacterial Infection	65	58	1.38 (0.97–1.98)
Dixon et al. (2006)	United Kingdom	Prospective Observational Study	9,018	Adalimumab, etanercept, infliximab	RA	6 months	58 years	7,664	1,354	Serious Infection	525	56	1.70 (1.29–2.26)
Carmona et al. (2007)	Spain	Registry For Active Long-Term Follow-Up	1,578	Infliximab, etanercept	RA	2 years	60	789	789	Serious Infection	114	63	1.54 (1.15–2.07)
										Malignancies	11	23	0.47 (0.23–0.97)
Bejarano et al. (2008)	United Kingdom	Multicenter, Randomized, Controlled Trial	148	Adalimumab	RA	1 year	47 ± 9	75	73	Serious Infection	13	11	1.18 (0.49–2.84)
Breedveld et al. (2006)	United States	Multicenter, Randomized, Double-Blind Clinical Trial	799	Adalimumab	RA	2 years	52 ± 14	274	257	Serious Infection	3	7	0.40 (0.10–1.55)
										Malignancies	2	4	0.47 (0.08–2.56)
										TB	1	0	2.82 (0.11–69.65)
Chen et al. (2009)	Taiwan	Randomized Double-Blind, Placebo-Controlled, Comparative Study	47	Adalimumab	RA	12 weeks	53	35	12	Adverse events	28	11	0.36 (0.04–3.31)
Detert et al. (2013)	Germany	Comparative Study	172	Adalimumab	RA	48 weeks	59.5	87	85	Serious Infection	3	4	0.72 (0.16–3.33)
										Malignancies	0	3	0.13 (0.01–2.65)
Kavanaugh et al. (2013)	United States	Randomised, Controlled Study	1,032	Adalimumab	RA	20 months	50.5	515	517	Serious Infection	13	6	2.21 (0.83–5.85)
										Malignancies	1	0	3.02 (0.12–74.24)
										TB	1	0	3.02 (0.12–74.24)
Huang et al. (2014)	China	Randomised, Controlled Trial	344	Adalimumab	AS	12 weeks	29.8	229	115	Serious Infection	1	0	1.52 (0.06–37.52)
Miyasaka (2008)	Japan	MulticenterDouble-Blind Study	352	Adalimumab	RA	24 weeks	54.4	265	87	Serious Infection	13	4	1.07 (0.34–3.37)
										Malignancies	0	2	0.06 (0.00–1.35)
Takeuchi et al. (2014)	Japan	Randomised, Double-Blind, Placebo-Controlled, Multicentre Study	334	Adalimumab	RA	70 days	54.0 ± 13.1	171	163	Serious Infection	2	1	1.92 (0.17–21.35)
Choy et al. (2012)	United Kingdom	Randomized, Double-Blind Study	247	Certolizumab pegol	RA	24 weeks	54.4	126	121	Serious Infection	3	2	-1.45 (0.24–8.84)
Fleischmann et al. (2009)	2009	Randomised Double-Blind Study	220	Certolizumab pegol	RA	24 weeks	53.8	111	109	Serious Infection	4	0	-9.17 (0.49–172.34)
Landewé et al. (2014)	Netherlands	A Double-Blind Randomised Placebo-Controlled phase 3 Study	325	Certolizumab pegol	AS	24 weeks	39.4	218	107	Serious Infection	2	0	-2.48 (0.12–52.17)
Mease et al. (2014)	United States	Double-Blind Randomised Placebo-Controlled Study	409	Certolizumab pegol	PsA	24 weeks	47.5	273	136	Serious Infection	4	1	2.01 (0.22–18.14)
Smolen et al. (2009)	United States	Multicentre, Randomised, Double-Blind, Placebo-Controlled, phase III Trial	461	Golimumab	RA	24 weeks	54.5	306	155	Serious Infection	4	3	0.67 (0.15–3.04)
										Malignancies	1	0	1.53 (0.06–37.70)
Weinblatt et al. (2003)	United States	Randomised Double-Blind Study	271	Adalimumab	RA	24 weeks	55.5	209	62	Serious Infection	2	0	1.51 (0.07–31.78)
										Malignancy	1	0	0.90 (0.04–22.35)
Galloway et al. (2013)	United Kingdom	Cohort Study	15,554	Etanercept, adalimumab, infliximab	RA	-	58	11,881	3673	Skin And Soft Tissue Infection	275	45	2.1428
Listing et al. (2005)	Germany	Case Control Study	1,529	Etanercept and infliximab	RA	12 months	55	928	601	Serious infections	200	39	3.96 (2.76–5.68)
										TB	1	0	1.95 (0.08–47.84)
Mercer et al. (2015)	United Kingdom	Prospective Cohort Study	15,016	Adalimumab, etanercept, infliximab	RA	3 years	58	11,767	3249	Cancer	239	93	-0.70 (0.55–0.90)
Axelrad et al. (2016)	United States	Retrospective Cohort Study	255	-	-	-	33	106	149	Cancer	22	46	-0.59 (0.33–1.05)
Scott et al. (2014)	United States	Cohort Study	6,841	Adalimumab, etanercept, infliximab, golimumab, certolizumab pegol	RA	12 months	-	932	5,909	Cancer	367	1,465	-1.97 (1.71–2.28)

### Meta-Analysis Results

#### Overall Adverse Effects

**FIGURE 2:**
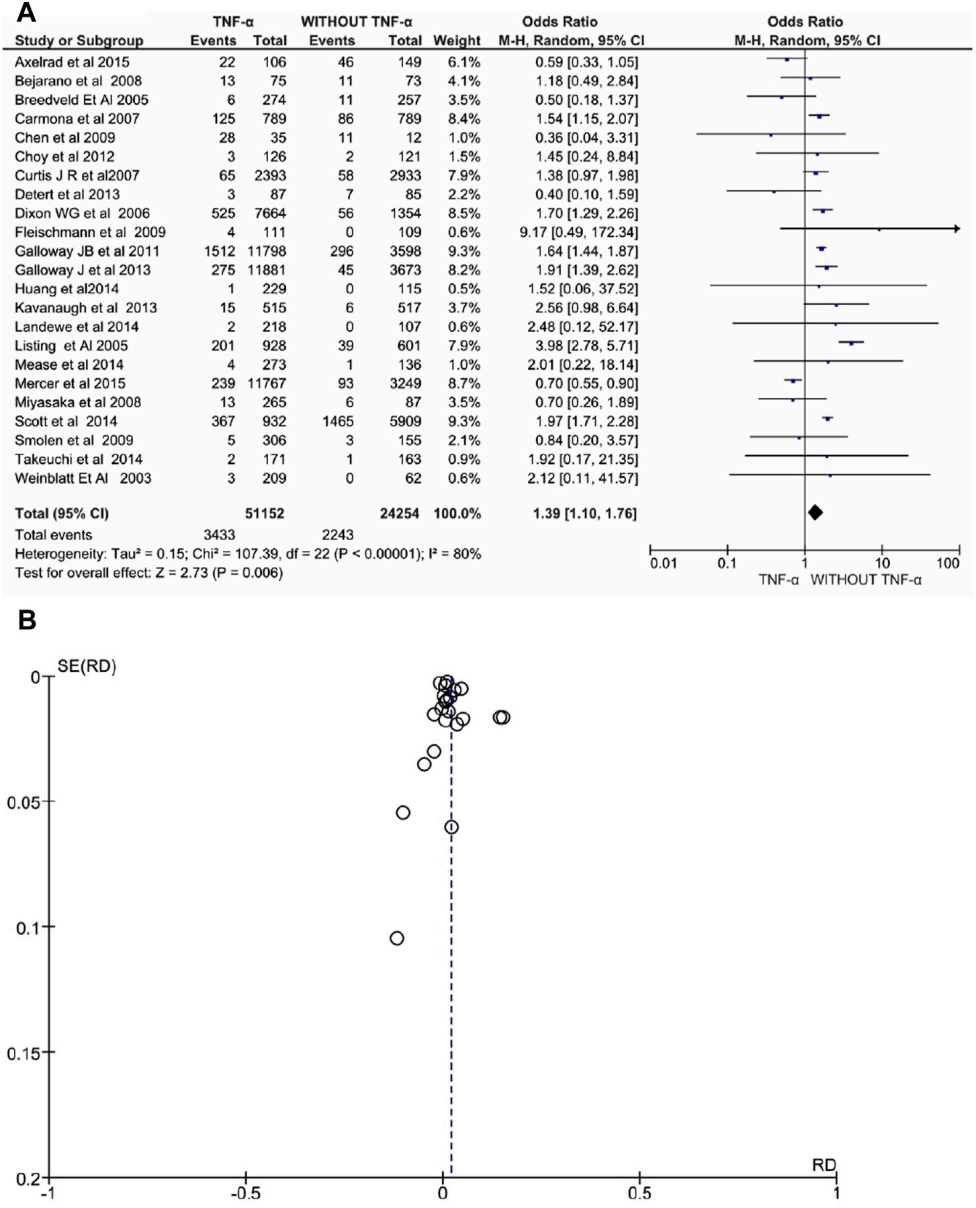
**(A)** Forest plot for overall adverse events after treatment with anti-TNFα agents versus control group (without anti-TNFα agents) using a random-effects model. Odds ratios and 95% confidence intervals are shown **(B)** Funnel plot for assessment of publication bias.

Twenty-three studies involving 75.406 patients with RA, PsA, or AS evaluated anti-TNFα drugs and reported a significant increase in overall adverse events. Exposure to anti-TNFα agents was associated with an increased risk of overall adverse events under the random-effects model (OR: 1.39, 95% CI: 1.10–1.76, *p* = 0.006, Figure 2). Additionally, the heterogeneity was high, *I* ^*2*^ = 80% (*p* < 0.00001). Finally, the funnel plot showed asymmetry, indicating a possible risk of publication bias.

#### Serious Infections

**FIGURE 3:**
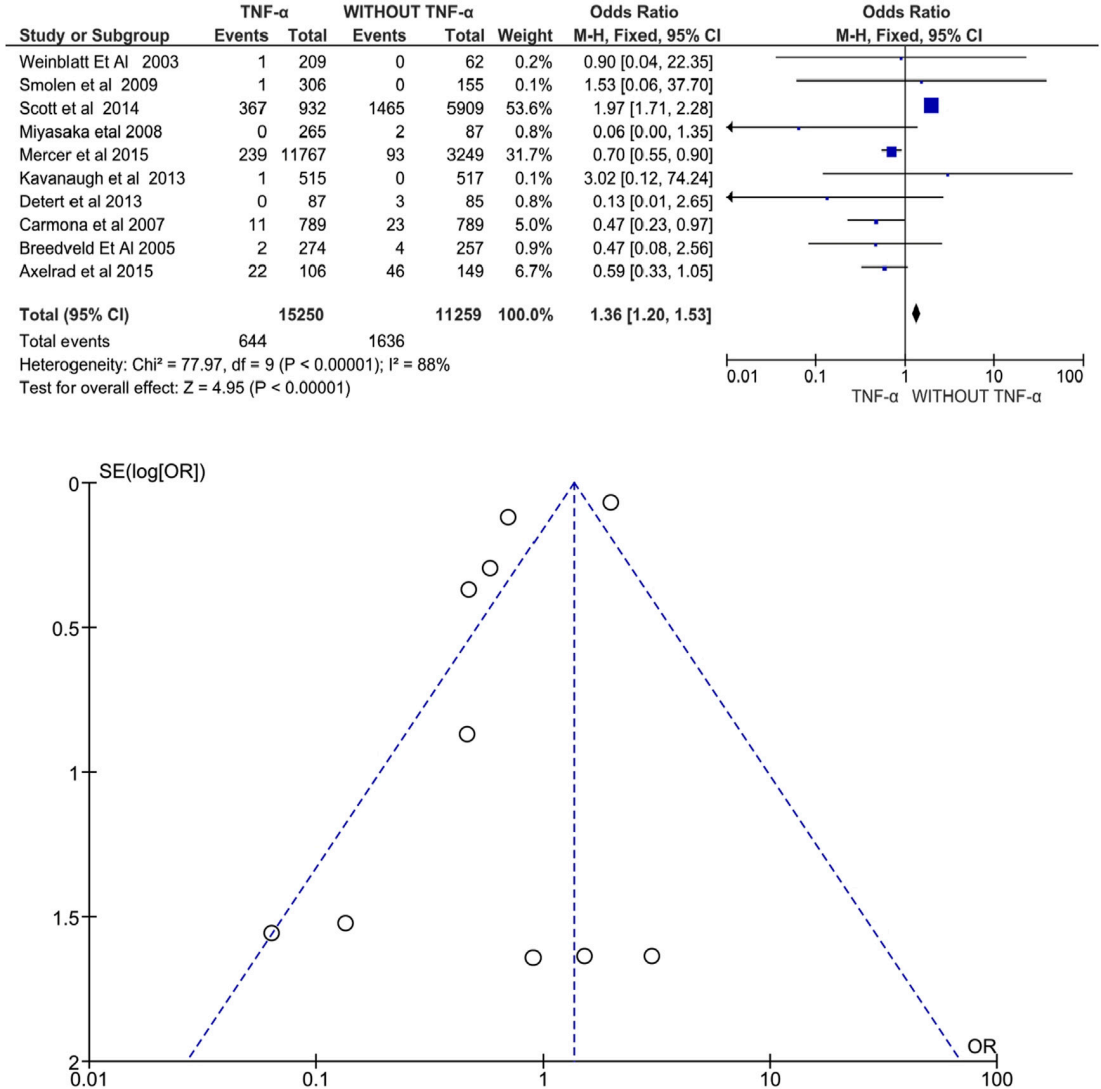
**(A)** Forest plot for serious infection after treatment with anti-TNFα agents versus control group (without anti-TNFα agents) using a random-effects model. Odds ratios and 95% confidence intervals are shown **(B)** Funnel plot for assessment of publication bias.

Eighteen studies involving 37,693 patients with RA, PsA, or AS evaluated anti-TNFα drugs and reported a significant increase in serious infections. Exposure to anti-TNFα agents was associated with an increased risk of serious infections under the random-effects model (OR: 1.72, 95% CI: 1.56–1.90, *p* < 0.00001, Figure 3). Additionally, the heterogeneity was moderate, *I* ^*2*^ = 49% (*p* = 0.01). The funnel plot showed asymmetry with the left corner of the pyramidal part of the funnel missing indicating a possible risk of publication bias.

#### Tuberculosis

**FIGURE 4:**
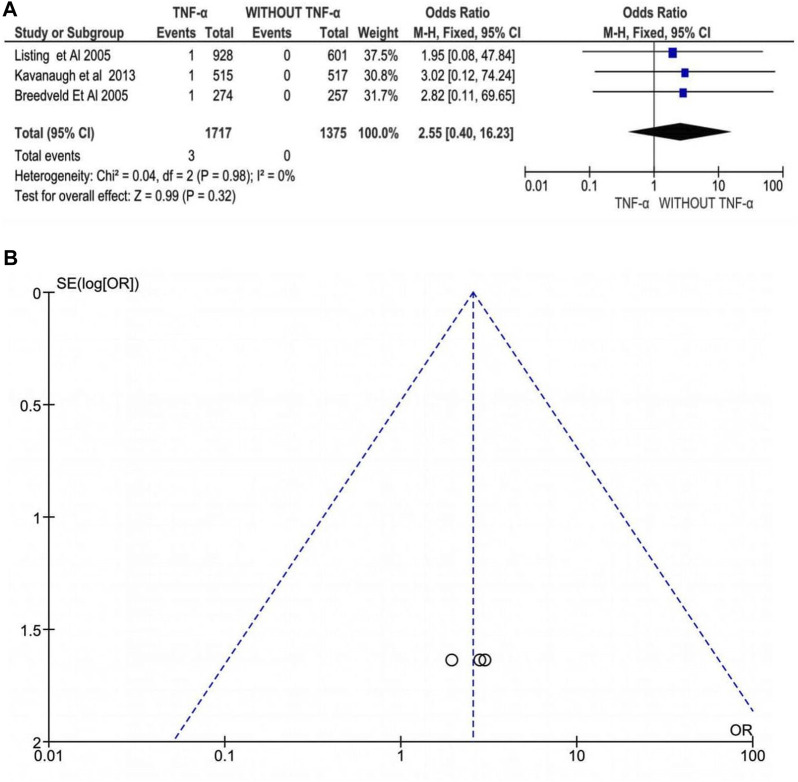
**(A)** Forest plot for tuberculosis infection after treatment with anti-TNFα agents versus control group (without anti-TNFα agents) using a random-effects model. Odds ratios and 95% confidence intervals are shown **(B)** Funnel plot for assessment of publication bias.

Three studies involving 3,092 patients with RA, PsA, or AS evaluated anti-TNFα drugs and reported an insignificant difference in the risk of tuberculosis following treatment with anti-TNFα agents. Exposure to anti-TNFα agents was not associated with an increased risk of tuberculosis under the random-effects model (OR: 2.55, 95% CI: 0.40–16.23, *p* = 0.32, Figure 4). Additionally, there was no heterogeneity between the studies, *I* ^*2*^ = 0% (*p* = 0.98). Thus, the funnel plot does not show significant publication bias, although the number of studies is too low to make a definitive conclusion.

#### Cancer

**FIGURE 5:**
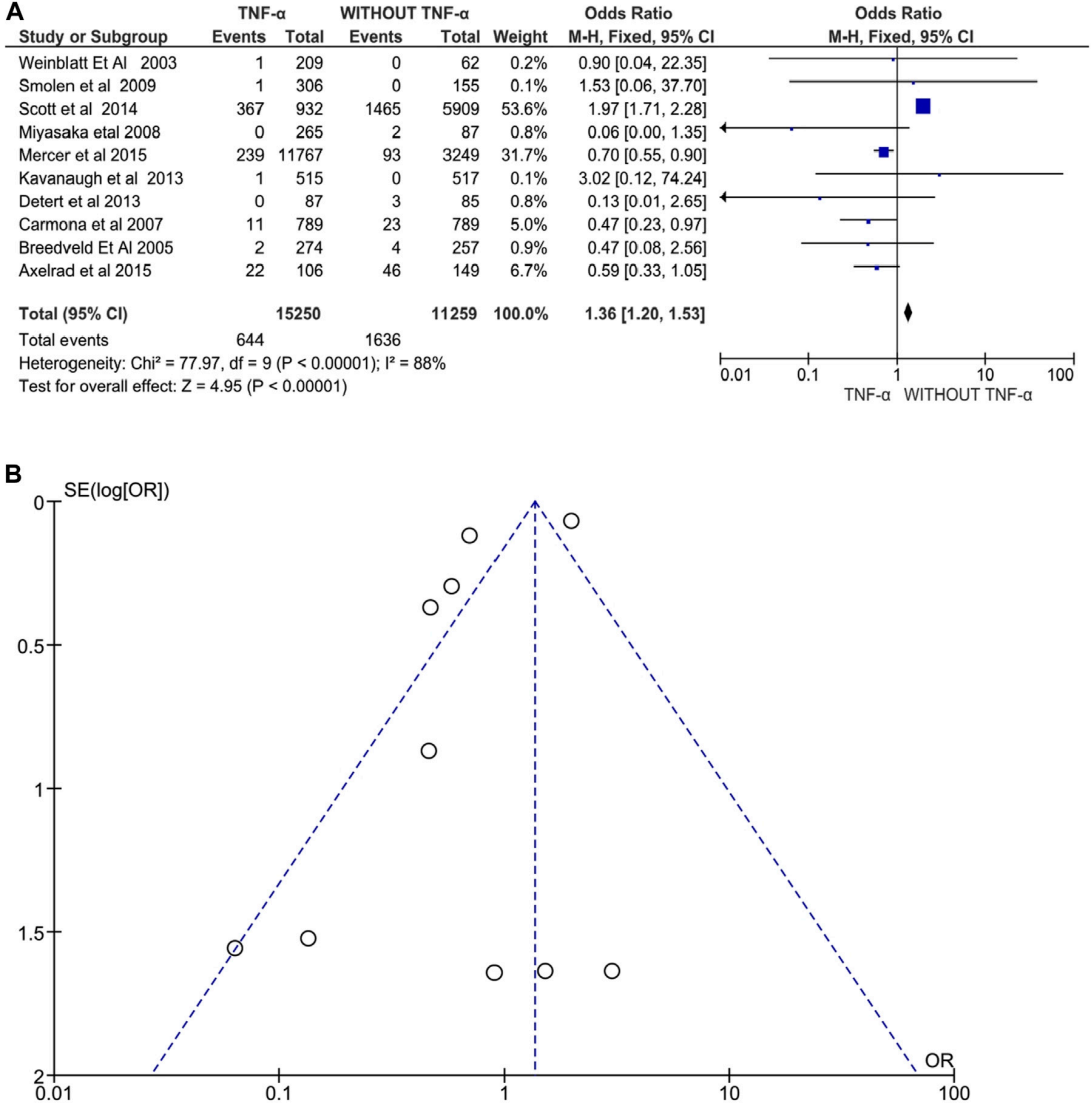
**(A)** Forest plot for cancer incidence after treatment with anti-TNFα agents versus control group (without anti-TNFα agents) using a random-effects model. Odds ratios and 95% confidence intervals are shown. **(B)** Funnel plot for assessment of publication bias.

Ten studies involving 26,509 patients with RA, PsA, or AS evaluated anti-TNFα drugs and reported a significant increase in cancer risk following treatment with anti-TNFα agents. Exposure to anti-TNFα agents was associated with an increased risk of cancer under the random-effects model (OR: 1.36, 95% CI: 1.20–1.53, *p* < 0.00001, Figure 5). Additionally, there was high heterogeneity between the studies, *I* ^*2*^ = 88% (*p* < 0.00001). The funnel plot does not show significant publication bias. Only three studies showed an increased risk of cancer development and were all conducted in patients with RA (Smolen et al., 2009; Scott et al., 2014 and Kavanaugh et al., 2013).

#### Risk of Bias

**FIGURE 6:**
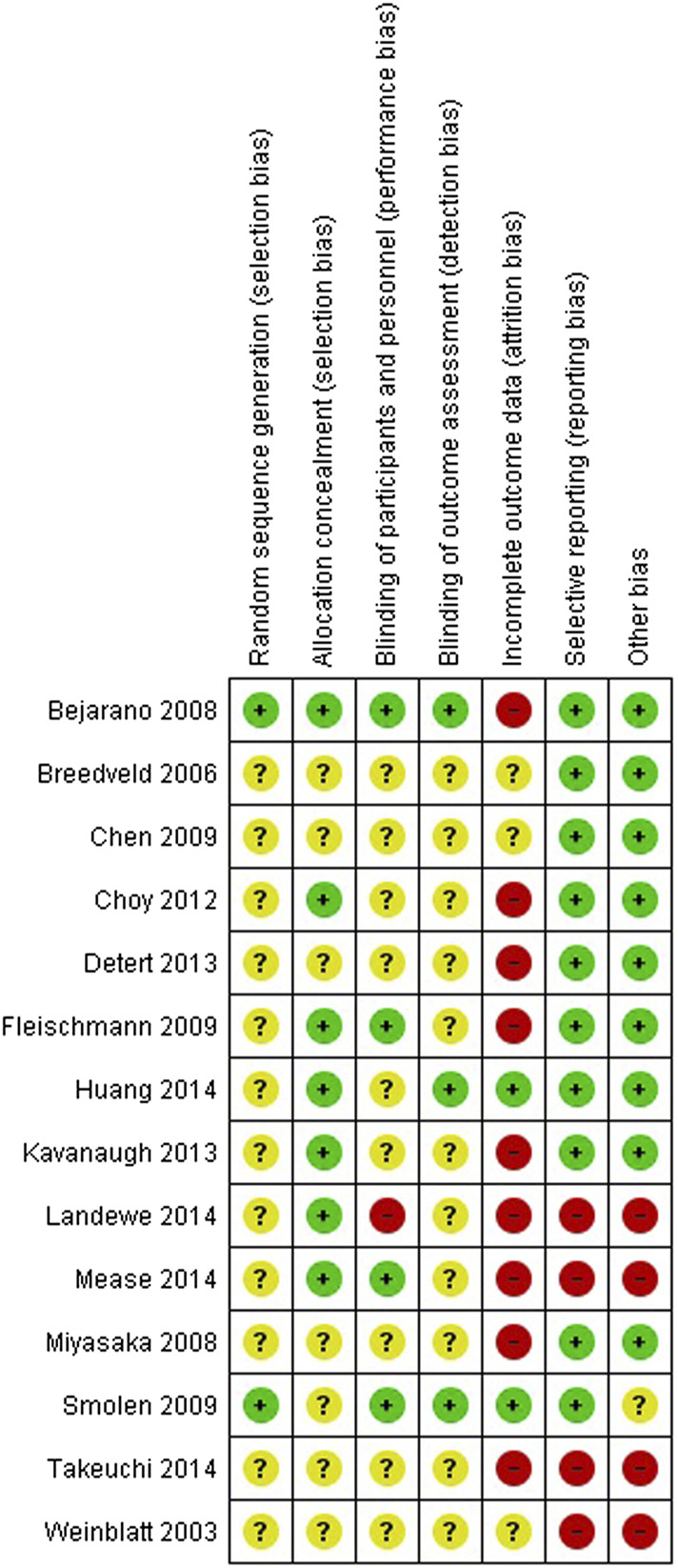
Risk of bias summary for randomized controlled trials and prospective studies included in the meta-analysis (+): low risk of bias (+): high risk of bias (?): unclear risk of bias.

The results of the risk of bias evaluation of the RCTs or comparative studies included in the meta-analysis are shown in Figure 6 (*n = 14*). Overall, there was a high risk of bias due to unclear or high risk due to randomization, selection, performance, and selection bias.

## Discussion

Tumor necrosis factor (TNF) is a pro-inflammatory agent formed in the macrophages, T cells, and synovial fibroblasts and is responsible for joint destruction and synovitis (Seymour et al., 2000). Elevation of TNF-α levels has been observed in synovial fluid and the synovium of patients with RA (Fütterer et al., 1998; Edwards et al., 2007). The development in biotechnology contributes to the development of enhanced biological agents, like anti-TNFα monoclonal antibodies, a potent treatment drug for chronic inflammatory diseases. However, the advancement of such drugs brings serious side effects along with its treatment potentials. The present meta-analysis was an effort to assess the various adverse effects after anti-TNF α therapy to treat rheumatoid arthritis, psoriatic arthritis, and ankylosing spondylitis. The findings of our meta-analyses pose questions about the application of anti-TNFα use, especially in infectious disease patients. The present meta-analysis showed a statistically significant increase in overall adverse events, serious infections, and malignancies associated with the use of anti-TNFα agents.

### Serious Infections

The present meta-analysis shows that there is an increased risk of various infections after treatment with anti-TNF agents. A case-control study carried out by Doran et al. (Doran et al., 2002) showed an increase in the risk of developing infections in patients with rheumatoid arthritis compared to non-RA subjects, particularly infections of the bone, joints, skin, soft tissues, and respiratory tract. The high frequency of infections in the RA group was attributed to the immunomodulatory effects of RA, or immunosuppressive agents used for RA treatment. Studies have also reported an increased risk of infections in older people, leukopenia, people on steroids, and smokers (Maini et al., 2004; Wolfe et al., 2006; Bernatsky et al., 2007; Edwards et al., 2007; Schneeweiss et al., 2007).

The Anti-TNF Trial in Rheumatoid Arthritis with Combination Therapy (ATTRACT) trial concluded that the frequency of serious infections was comparable between those that received MTX/infliximab and those treated with MTX38. A similar trial by Goekoop-Ruiterman YP et al. found an increase in the risk of serious infections following treatment with anti TNF agents (Goekoop-Ruiterman et al., 2005)Schneeweiss et al. performed a prospective cohort study and noted that the risk of serious bacterial infections in those treated with anti-TNF agents was high compared to users of DMARDs and MTX (Schneeweiss et al., 2007).

Listing et al. conducted a cohort study and reported a two times higher risk of serious upper and lower respiratory tract infection in patients treated with etanercept and infliximab (Listing et al., 2005). Similarly, the present meta-analysis observed that the odds of serious infection were 1.72 times higher in the anti-TNFα agent treatment group (95% CI: 1.56–1.90, *p* < 0.00001). A moderate heterogeneity of *I* ^*2*^ = 49% can be attributed to multiple inflammatory conditions and different anti-TNFα agents and comparators that were all pooled together in the same analysis.

Contrastingly, a meta-analysis of randomized controlled trials of the safety of TNF blockers in over 8,800 RA patients did not identify an increased risk of serious bacterial infection in the standard recommended dose. However, a dose-response increase in sepsis was observed with high dose biological therapy^−^ (Leombruno et al., 2009). Another meta-analysis by Alonso-Ruiz et al. (2008) showed an increased risk of serious infections upon treatment with adalimumab and etanercept versus controls.

### Tuberculosis

It is critical to develop an effective latent tuberculosis infection screening technique until starting anti-TNF therapy in patients with immune-mediated inflammatory diseases. Therefore, implementing guidelines for latent tuberculosis infection screening and (prophylactic) treatment before starting anti-TNF therapy could lower infection rates. The tuberculosis prevalence rate was consistently higher for all anti-TNF drugs than in the general population, with infliximab and adalimumab showing higher rates compared to etanercept. Monoclonal anti-TNF α such as infliximab or adalimumab antibodies pose a higher risk of TB incidence than soluble TNF-α receptor, etanercept due to differential abilities to bind to the TNF receptor. Studies have found that apoptosis occurs upon binding of infliximab to membrane TNF on T cells and monocytes which may lead to reduction in number of antimycobacterial effector cells and/or dissolution of granulomas. In contrast, etanercept does not cause apopotosis of cells that express membrane TNF (Minozzi et al., 2016). Our results showed a non-significant difference in the incidence of tuberculosis following treatment with anti-TNFα agents (OR: 2.55, 95% CI: 0.40–16.23, *p* = 0.32). However, the number of studies included was low (*n = 3* studies), making it necessary to interpret the results cautiously as they may not be reliable.

Various biologic registries have shown an increased risk of TB infection in patients treated with monoclonal antibodies than TNF blockers (Leslie et al., 2013). The Brazilian Society of Rheumatology’s guidelines states that all patients should have baseline chest X-ray and tuberculin skin test (PPD) before treatment with biologic DMARDs (Da Mota et al., 2012). They have also prescribed a 6-month course of isoniazid a month before anti-TNFα therapy in patients with a PPD of ≥5 mm with the previous TB on chest X-ray or who have had contact with tuberculosis patients. Strict adherence to guidelines for prescribing TNF-α blockers led to a decrease in tuberculosis’s incidence rate ratios to that of the normal-population (Bongartz et al., 2006).

### Cancer Risk

According to Bongartz et al., the risk of malignancy increases three times when treated with infliximab and adalimumab (Bongartz et al., 2006)*.* Similarly, the present meta-analysis observed that the odds of cancer were 1.36 times higher in the anti-TNFα agent treatment group (95% CI: 1.20–1.53, *p* < 0.00001). High heterogeneity of *I* ^*2*^ = 88% can be attributed to multiple inflammatory conditions and different anti-TNFα agents and comparators that were all pooled together in the same analysis. The cancer risk is higher in three of 10 studies, namely Smolen et al., 2009, Scott et al., 2014, and Kavanaugh et al., 2013 which were all conducted in patients with RA. In contrast, Bonovas et al. have reported no significant effect of anti-TNF agents (adalimumab, golimumab, infliximab, certolizumab, or etanercept) on cancer risk in adult patients with rheumatologic disease. However, this meta-analysis indicates the risk of publication bias *via* funnel plot asymmetry suggesting an overestimation of the pooled risk for cancer.

## Limitations

Although the present meta-analyses raise concerns about the use of anti-TNFα agents to treat inflammatory conditions based on higher incidences of serious infections and cancer, it is important to interpret these results with caution. The number of studies is limited, and the event rate is low, particularly regarding the development of tuberculosis, raising concern about the reliability of the pooled estimate. The risk of bias was unclear to high across most domains in the included RCTs, limiting the results obtained. Further, the development of specific adverse events such as cancer may not have been appropriately captured in the follow-up period of most studies leading to a possible underestimation of the true odds ratio. Subgroup analysis by anti-TNFα agent type, comparator, or inflammatory condition would also address high to moderate heterogeneity observed for some of the meta-analysis results.

## Conclusion

Synthesis of current evidence from RCTs, data registries, and prospective studies involving the use of anti-TNFα agents for the treatment of inflammatory conditions such as rheumatoid arthritis, psoriatic arthritis, and ankylosing spondylitis suggests an increased risk of serious infections and malignancies. However, long-term surveillance and monitoring of patients on anti-TNFα treatment *via* data registries and long-term epidemiological studies are necessary to capture any long-term complications, particularly the development of cancers that can occur long after the follow-up time of RCTs.

## Data Availability

The raw data supporting the conclusions of this article will be made available by the authors, without undue reservation.

## Abbreviations

TNF, Tumor Necrosis Factor; RA, Rheumatoid Arthritis.
